# Receipt of medications for opioid use disorders among veterans by race/ethnicity and legal involvement: an observational study of electronic health records

**DOI:** 10.1186/s40352-025-00336-6

**Published:** 2025-04-29

**Authors:** Andrea K. Finlay, Ekaterina Pivovarova, Mengfei Yu, Christine Timko, Ingrid A. Binswanger, David Smelson, Emmeline Taylor, Alex H. S. Harris

**Affiliations:** 1 VA Palo Alto Health Care System, Menlo Park, USA; 2 UMass Chan Medical School, Worcester, USA; 3 Kaiser Permanente Colorado, Aurora, USA; 4 University of Colorado School of Medicine, Aurora, USA; 5 Colorado Permanente Medical Group, Denver, USA; 6 Stanford University School of Medicine, Stanford, USA; 7 Edith Nourse Rogers Memorial Veterans Hospital, Bedford, USA; 8 University of Colorado Colorado Springs, Colorado Springs, USA; 9 Kaiser Permanente Bernard J. Tyson School of Medicine, Pasadena, USA

**Keywords:** Opioid-related disorders, Methadone, Buprenorphine, Naltrexone, United States Department of Veterans Affairs

## Abstract

**Background:**

The Veterans Health Administration has made strides to improve access to medications for opioid use disorder overall. However, quality improvement methods to assess treatment gaps may not sufficiently detect differences in medication access by intersecting patient factors, which may have multiplicative rather than additive effects. This study aimed to determine whether race/ethnicity and legal involvement interact in receipt of medications for opioid use disorder among Veterans Health Administration patients.

**Methods:**

Using national electronic health record data from Fiscal Years 2021–2022, we examined the receipt of medications for opioid use disorder among veterans diagnosed with opioid use disorder who received healthcare at Veterans Health Administration facilities (*n* = 65,883). We conducted a mixed effects multivariable logistic regression model to examine an interaction effect of race/ethnicity and legal involvement with receipt of any medications for opioid use disorder, both unadjusted and adjusted for patient and facility characteristics.

**Results:**

In an adjusted logistic regression model, the interaction effect indicated that non-Hispanic Black veterans with legal involvement had the lowest odds of medications for opioid use disorder receipt compared to non-Hispanic White veterans without legal involvement (adjusted odds ratio = 0.67, 95% confidence interval = 0.59–0.77, *p* <.0001). Non-Hispanic American Indian/Alaska Native patients without legal involvement (adjusted odds ratio = 0.85, 95% confidence interval = 0.73–0.99, *p* =.04) also had lower odds of receipt of medications for opioid use disorder compared to non-Hispanic White patients without legal involvement. Non-Hispanic White veterans with legal involvement (adjusted odds ratio = 1.07, 95% confidence interval = 1.01–1.14, *p* =.03) had higher odds of receipt of medications for opioid use disorder compared to non-Hispanic White patients without legal involvement.

**Conclusions:**

Veterans Health Administration quality improvement efforts should monitor interacting racial and legal status factors and understand and address patient, clinical, and regulatory barriers to medications for opioid use disorder among Black veterans with legal involvement.

## Background

The United States (US) opioid epidemic remains a national public health crisis and veterans have been particularly affected with opioid overdose rates that steadily rose from 2010 to 2016 (Lin et al., 2019). Among US Veterans Health Administration (VHA) patients, those with a legal history, defined as contact with the Veterans Justice Programs that provide outreach to veterans in court, jail, and prison settings, had 38% higher odds of opioid overdose over a four-year period compared to patients without a legal history (Finlay et al., 2022). US veterans and non-veterans from racial/ethnic minority backgrounds, such as Black and Hispanic, have been over-represented in the criminal legal system (Pettit & Gutierrez, 2018; Tsai et al., 2013). Overdose deaths involving any drug and fentanyl increased among most racial and ethnic groups from 2018 to 2021 (Han et al., 2022). Among people ages 35 to 64, non-Hispanic Black men and American Indian or Alaskan Native men had the highest overall drug overdose rates and Black men had the highest fentanyl-involved overdose death rates.

Medications for opioid use disorder (MOUD; i.e., methadone, buprenorphine, and extended-release naltrexone) are evidence-based treatments for opioid use disorder and methadone and buprenorphine reduce opioid-related deaths (Department of Veterans Affairs & Department of Defense, 2021; Volkow et al., 2019). Within the VHA, it has been a high priority to improve use of MOUD and there have been ongoing efforts to increase prescribing of MOUD since 2006 (Wyse et al., 2018). Overall, 41% of VHA patients diagnosed with opioid use disorder received one or more types of MOUD in Fiscal Year 2017 (Finlay et al., 2021). However, there was tremendous variation across the VHA system by location and patient population. Non-Hispanic Black patients, compared to White patients, and patients with legal involvement, compared to patients with no legal involvement, at the VHA, had 0.89 and 0.80 lower odds of receiving MOUD, respectively (Finlay et al., 2021). However, research examining the interaction between race and legal involvement is needed to ensure that health differences are considered in access to and engagement in treatment programs (Mark et al., 2023).

Studying the interaction of race and legal status among veterans is important because of known variation by age, race/ethnicity, and sex in drug overdose deaths and lack of information on why these differences exist. Variation in access to MOUD for by racial/ethnic group or legal status may suggest an interaction effect. For example, Black veterans are disproportionately incarcerated compared to White veterans (Tsai et al., 2013) and MOUD availability is limited in prisons and jails (Scott et al., 2021; Sufrin et al., 2023). Black veterans with legal involvement may have fewer opportunities to learn about or initiate MOUD treatment than White veterans. Similarly, Black people arrested on drug charges had fewer opportunities to connect with drug diversion treatment than White people (Nicosia et al., 2013). When given a choice of medications while incarcerated Black patients were more likely to choose buprenorphine than White patients (Berk et al., 2025), but in the community buprenorphine is less often prescribed to Black patients compared to White patients (Lagisetty et al., 2019), suggesting that care may be disrupted for Black patients with legal involvement once they return to their communities. Black patients are also likely to receive lower dosages of methadone and buprenorphine as compared to non-Hispanic White patients in the community, suggesting that even when in agonist treatment, Black patients may be undertreated (Ross et al., 2024). Black patients also have more distrust of MOUD and the healthcare system than White patients and more often experience criminalization of opioid use (Cuevas et al., 2016; Husain et al., 2023; James & Jordan, 2018; Saha et al., 2008), which may further deter them from initiating these medications if they fear becoming re-involved in the legal system.

Although the VHA has made strides to improve access to MOUD overall (Wyse et al., 2018), quality improvement methods to assess gaps in treatment access may not sufficiently detect differences in medication access by interacting patient factors, which may have multiplicative rather than additive effects. The goal of this study was to examine the interaction of race/ethnicity and legal involvement in receipt of MOUD among VHA patients. We hypothesized that patients who were both from racial/ethnic minority groups, such as Black or Hispanic, and legal involved (race * legal involvement) would have lower receipt of MOUD than patients who are non-Hispanic White with no legal involvement.

## Methods

### Data source and participants

Data were from the VHA Corporate Data Warehouse, which includes all records of veteran patients enrolled in the VHA. Participants included veteran patients who received an opioid use disorder diagnosis during an outpatient or inpatient visit in Fiscal Years 2021 through 2022 (October 1, 2020 through September 30, 2022). The earliest diagnosis record during the observation period was used. This study was approved by the Stanford University Institutional Review Board (#44494) and the VA Palo Alto Research & Development Committee in accordance with the Declaration of Helsinki. All data reported in this manuscript were approved under a waiver of consent.

### Measures

#### Receipt of any medications for opioid use disorder

The primary outcome measured was receipt of any MOUD, defined following the Version 2 American Society for Addiction Medicine’s (ASAM) specifications (Harris et al., 2016). Patients who attended at least one methadone outpatient clinic with a concurrent opioid use disorder diagnosis and/or filled at least one prescription for buprenorphine (oral or injectable) or buprenorphine/naloxone (sublingual) or naltrexone (oral or injectable) during the same Fiscal Year (2021 or 2022) as their opioid use disorder diagnosis were coded as having received MOUD. Veterans with a VA or VA-contracted methadone clinic visit and concurrent opioid use disorder diagnosis were assumed to have received methadone or buprenorphine at the visit. Veterans with a methadone clinic visit and concurrent cancer diagnosis were excluded to ensure methadone receipt was not for pain management. Buprenorphine or naltrexone prescription fills were identified from VA pharmacy records. All facilities had a record of a buprenorphine and all but one facility had a record of a naltrexone prescription fill indicating that these medications were offered at almost all facilities. The ASAM specifications for MOUD (Harris et al., 2016) were used in this study because these standard specifications were defined and developed using VHA data sources. ASAM specifications are conceptually similar to the US Centers for Medicare & Medicaid Services Use of Pharmacotherapy for Opioid Use Disorder quality measure (Center for Medicaid and CHIP Services, Centers for Medicare & Medicaid Services, 2022).

### Race/ethnicity and legal involvement

The primary independent variables were race/ethnicity and legal involvement. Race and ethnicity were drawn from the patient record and coded as Hispanic, non-Hispanic American Indian/Alaskan Native, non-Hispanic Asian (including Native Hawaiian/Pacific Islander), non-Hispanic Black, or non-Hispanic White. We examined the distribution of racial groups within the Hispanic category, but because of small sample sizes were unable to analyze them separately.

Patients were coded as with legal involvement if they had an encounter with the VA Veterans Justice Programs as indicated by an outpatient clinic code or Homeless Operations Management and Evaluation System record (Blue-Howells et al., 2013). Outreach is largely focused on veterans with active or recent criminal legal involvement; therefore, we identified veterans with legal involvement if their encounter with the Veterans Justice Programs occurred within the current or prior Fiscal Year.

The mission of the Veterans Justice Programs is to provide outreach to veterans in criminal legal settings, including courts, jails, and prisons and link them to indicated healthcare, housing and other services (Blue-Howells et al., 2013). Veterans who had an encounter with Veterans Justice Programs received outreach from VHA staff while involved with the criminal legal system, including while incarcerated in prison or jail, involved in court proceedings, or during or after arrest. We were not able to identify veterans who were involved in the legal system but did not have contact with Veterans Justice Programs. Although VHA staff cannot provide treatment while veterans are incarcerated, outreach specialists meet with veterans in prison or jail, assess their treatment needs including for opioid use disorder, and link veterans after release to VHA and community substance use disorder treatment clinics and other clinics where they can initiation MOUD, if indicated and desired. Veterans with opioid use may be connected to overdose education resources as well.

### Patient and facility characteristics

We included patient and facility characteristics that were associated with receipt of MOUD in prior studies or were clinically relevant (Finlay et al., 2016a, 2021; Knudsen & Roman, 2009). Patient socio-demographic characteristics included sex (female, male; from the electronic health record), age, marital status, rural or urban residence (defined by the U.S. Census Bureau) (Ratcliffe et al., 2016), and housing status (defined by International Classification of Diseases-10 codes for homelessness, utilization of VHA homeless services [clinic and bed section codes], and a homeless indicator variable [from a health factors list]) (Finlay et al., 2016). Patient health characteristics included co-occurring mental health diagnoses, co-occurring substance use disorder diagnoses, the Deyo comorbidity index coded as any versus no comorbidities (Deyo et al., 1992), and service-connected disability rating, which is based on conditions caused or exacerbated by military service.

Facility characteristics included total number of patients at that facility, the proportion of patients with an opioid use disorder diagnosis at that facility (the number of patients with an opioid use disorder diagnosis divided by the total number of patients), the proportion of women patients at that facility (the number of women patients divided by the total number of patients), and proportion of racial/ethnic minority patients at that facility (the number of Hispanic, non-Hispanic American Indian/Alaskan Native, non-Hispanic Asian, and non-Hispanic Black patients divided by the total number of patients). Each facility location was coded as rural or urban and coded by geographic region (i.e., Northeast, South, Midwest, West). Substance use disorder treatment services available at the facility included inpatient withdrawal management, substance use disorder residential treatment, methadone clinic, and percentage of patients who received medications for alcohol use disorder. The focus of this study is on race/ethnicity and legal involvement; therefore, all other patient and facility characteristics were included as controls in the statistical models.

### Statistical analysis

Patients who were diagnosed with opioid use disorder, but who were missing data (*n* = 1350; 2%) were excluded. Descriptive statistics were reported for the total sample and stratified by legal involvement. Differences by legal status were tested using chi-square or t-tests. To examine the association of race/ethnicity and legal involvement with receipt of any MOUD, we conducted a mixed effects multivariable logistic regression model, both unadjusted and adjusted for patient and facility characteristics. To account for clustering, the regression model included a random effect for the facility (*n* = 129) where a veteran received their opioid use disorder diagnosis. We then used the model to produce the predicted population margin of MOUD receipt and 95% confidence intervals among patients in each race/ethnicity by legal involvement group. All analyses were conducted using SAS software version 9.04 (SAS Institute Inc., 2013). We considered a 2-sided *p* <.05 to be statistically significant.

## Results

### Sample characteristics

A total of *N* = 65,883 patients at 129 VHA facilities in Fiscal Years 2021–2022 were diagnosed with opioid use disorder (Table 1). The sample was 72.5% non-Hispanic White (*n* = 47,748), 10.5% with legal involvement (*n* = 6,908), 8.0% women (*n* = 5,298), with a mean age of 54.9 (standard deviation [SD] = 14.6) years. Co-occurring mental health disorders (patients with legal involvement: 6,039/6,908 [87.4%] and without legal involvement 42,680/58,975 [72.4%]), co-occurring substance use disorders (5,579/6,908 [80.8%] and 27,474/58,975 [46.6%], respectively), and homelessness or participation in a VHA homeless program during the observation period (4,959/6,908 [71.8%] and 24,695/58,975 [41.9%], respectively) were common among both patients groups, but significantly more common among veterans with legal involvement (*p* <.001). Sample characteristics are reported overall and by legal status in Table 1.

**Table 1:** Veterans with opioid use disorders in fiscal years 2021–2022, overall and stratified by legal involvement

	Overall Sample	Veterans With Legal involvement	Veterans Without Legal Involvement	
	N (%)	N (%)	N (%)	*p*
Sample size	65,883 (100)	6,908 (10.5)	58,975 (89.5)	
Race/Ethnicity				< 0.0001
Non-Hispanic American Indian/ Alaskan Native	867 (1.3)	108 (1.6)	759 (1.3)	
Non-Hispanic Asian	775 (1.2)	88 (1.3)	687 (1.2)	
Non-Hispanic Black	12,606 (19.1)	1,156 (16.7)	11,450 (19.4)	
Hispanic	3,887 (5.9)	403 (5.8)	3,484 (5.9)	
Non-Hispanic White	47,748 (72.5)	5,153 (74.6)	42,595 (72.2)	
Sex				0.088
Female	5,298 (8.0)	519 (7.5)	4,779 (8.1)	
Male	60,585 (92.0)	6,389 (92.5)	54,196 (91.9)	
Age – mean (SD)	54.9 (14.6)	45.3 (12.6)	56.0 (14.4)	< 0.0001
Marital status				< 0.0001
Married	22,356 (33.9)	1,488 (21.5)	20,868 (35.4)	
Not married	43,527 (66.1)	5,420 (78.5)	38,107 (64.6)	
Residence				< 0.0001
Rural	17,070 (25.9)	1,579 (22.9)	15,491 (26.3)	
Urban	48,813 (74.1)	5,329 (77.1)	43,484 (73.7)	
Housing status				< 0.0001
Homeless	29,654 (45.0)	4,959 (71.8)	24,695 (41.9)	
Housed	36,229 (55.0)	1,949 (28.2)	34,280 (58.1)	
Co-occurring mental health diagnosis	48,719 (74.0)	6,039 (87.4)	42,680 (72.4)	< 0.0001
Co-occurring substance use disorder diagnosis	33,053 (50.2)	5,579 (80.8)	27,474 (46.6)	< 0.0001
Deyo co-morbidity index	7,737 (11.7)	402 (5.8)	7,335 (12.4)	< 0.0001
Service-connected disability rating				< 0.0001
None	22,737 (34.5)	1,874 (27.1)	20,863 (35.4)	
<50%	9,086 (13.8)	985 (14.3)	8,101 (13.7)	
≥50%	34,060 (51.7)	4,049 (58.6)	30,011 (50.9)	

SD = standard deviation

### Receipt of medications for opioid use disorder

The overall raw rate of receipt of MOUD was 41.7%. The raw rate of receipt of MOUD by race/ethnicity, legal status, and the intersection of these characteristics is reported in Table 2. For race/ethnicity, the rate of receipt ranged from 36.3% (*n* = 315 of 867; 95% confidence interval [CI] = 33.1–39.5%) for non-Hispanic American Indian/Alaskan Native veterans to 42.7% (*n* = 1,661 of 3,887; 95% CI = 41.2–44.3%) for Hispanic veterans. In the unadjusted logistic regression model, odds for receipt of MOUD was significantly lower for non-Hispanic Black veterans (odds ratio [OR] = 0.63, 95% CI = 0.59–0.68, *p* <.0001) compared to non-Hispanic White veterans. Veterans with legal involvement had higher odds of receipt of MOUD compared to Veterans without legal involvement (OR = 1.35, 95% CI = 1.18–1.55, *p* <.0001). The interaction effect indicated that non-Hispanic Black veterans with legal involvement (OR = 0.74, 95% CI = 0.65–0.84, *p* <.0001) and without legal involvement (OR = 0.82, 95% CI = 0.79–0.86, *p* <.0001) had lower odds of receipt of MOUD compared to non-Hispanic White veterans without legal involvement. Non-Hispanic Asian veterans with legal involvement (OR = 1.67, 95% CI = 1.09–2.56, *p* =.02), Hispanic veterans with legal involvement (OR = 1.41, 95% CI = 1.15–1.72, *p* =.001) and without legal involvement (OR = 1.08, 95% CI = 1.00-1.17, *p* =.047), and non-Hispanic White veterans with legal involvement (OR = 1.53, 95% CI = 1.44–1.62, *p* <.0001) had higher odds of receipt of MOUD compared to non-Hispanic White veterans without legal involvement.

**Table 2:** Receipt of medications for opioid use disorder among veterans in fiscal years 2021–2022

	Raw Rate of MOUD Receipt	Unadjusted MOUD Receipt	Adjusted MOUD Receipt	Predicted MOUD Receipt
	n/N (%) [95% CI]	OR (95% CI)	AOR (95% CI)	Predicted Population Margin (95% CI)
Race/Ethnicity				
Non-Hispanic American Indian/ Alaskan Native	315/867 (36.3) [33.1–39.5]	0.83 (0.67–1.02)	0.83 (0.66–1.02)	38.3 (33.0-43.8)
Non-Hispanic Asian	313/775 (40.4) [36.9–43.8]	1.00 (0.79–1.26)	0.96 (0.76–1.21)	41.9 (36.0–48.0)
Non-Hispanic Black	4,998/12,606 (39.6) [38.8–40.5]	0.63*** (0.59–0.68)	0.78*** (0.72–0.84)	36.9 (34.4–39.5)
Hispanic	1,661/3,887 (42.7) [41.2–44.3]	1.00 (0.89–1.12)	0.96 (0.85–1.07)	41.8 (38.5–45.2)
Non-Hispanic White	20,189/47,748 (42.3) [41.8–42.7]	Ref	Ref	42.9 (40.7–45.1)
Legal status				
With legal involvement	3,366/6,908 (48.7) [47.5–49.9]	1.35*** (1.18–1.55)	0.98 (0.85–1.12)	40.0 (36.4–43.8)
Without legal involvement	24,110/58,975 (40.9) [40.5–41.3]	Ref	Ref	40.6 (38.3–43.0)
Race/Ethnicity * Legal status				
Non-Hispanic American Indian/ Alaskan Native * With legal involvement	45/108 (41.7) [32.4–51.0]	1.20 (0.81–1.78)	0.86 (0.57–1.28)	38.3 (29.3–48.3)
Non-Hispanic Asian * With legal involvement	46/88 (52.3) [41.8–62.7]	1.67* (1.09–2.56)	1.12 (0.72–1.73)	44.8 (34.2–55.8)
Non-Hispanic Black & With legal involvement	420/1,156 (36.3) [33.6–39.1]	0.74*** (0.65–0.84)	0.67*** (0.59–0.77)	32.9 (29.6–36.3)
Hispanic & with legal involvement	193/403 (47.9) [33.6–52.8]	1.41* (1.15–1.72)	0.95 (0.77–1.17)	40.9 (35.6–46.3)
Non-Hispanic White * With legal involvement	2,662/5,153 (51.7) [50.3–53.0]	1.53*** (1.44–1.62)	1.07* (1.01–1.14)	43.7 (41.2–46.3)
Non-Hispanic American Indian/ Alaskan Native * Without legal involvement	270/759 (35.6) [32.2–39.0]	0.87 (0.74–1.01)	0.85* (0.73–0.99)	38.2 (34.1–42.5)
Non-Hispanic Asian * Without legal involvement	267/687 (38.9) [35.2–42.5]	0.92 (0.78–1.07)	0.88 (0.75–1.04)	39.0 (34.8–43.4)
Non-Hispanic Black * Without legal involvement	4,578/11,450 (40.0) [39.1–40.9]	0.82*** (0.79–0.86)	0.96 (0.92–1.01)	41.1 (38.8–43.5)
Hispanic & Without legal involvement	1,468/3,484 (42.1) [40.5–43.8]	1.08* (1.00-1.17)	1.03 (0.95–1.11)	42.8 (40.0-45.6)
Non-Hispanic White * Without legal involvement	17,527/42,595 (41.1) [40.7–41.6]	Ref	Ref	42.1 (39.9–42.2)

MOUD = medications for opioid use disorder. OR = odds ratio. CI = confidence interval. AOR = adjusted odds ratio

*p <.05. ***p <.001

The adjusted logistic regression model of MOUD receipt by race and legal status, controlling for patient and facility characteristics, is reported in Table 2. Odds of receipt of MOUD was significantly lower for non-Hispanic Black patients compared to non-Hispanic White patients (adjusted odds ratio [AOR] = 0.78, 95% CI = 0.72–0.84, *p* <.0001). The interaction effect indicated that non-Hispanic Black patients with legal involvement (AOR = 0.67, 95% CI = 0.59–0.77, *p* <.0001) had lower odds of MOUD receipt compared to the reference group of non-Hispanic White patients without legal involvement. Non-Hispanic American Indian/Alaskan Native veterans without legal involvement also had lower odds of receipt of MOUD compared to non-Hispanic White veterans without legal involvement (AOR = 0.85, 95% CI = 0.73–0.99, *p* =.04). Non-Hispanic White veterans with legal involvement had higher odds of receipt of MOUD compared to non-Hispanic White veterans without legal involvement (AOR = 1.07, 95% CI = 1.01–1.14, *p* =.03).

**Fig. 1:**
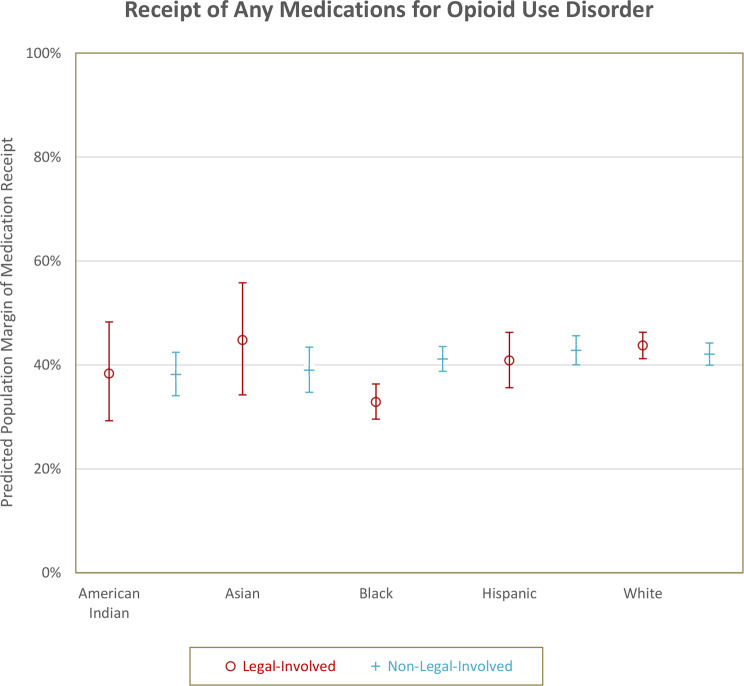
Predicted population margin of medications for opioid use disorder receipt by race/ethnicity and legal status

The predicted population margin of MOUD receipt and 95% confidence intervals by race and legal status are reported in Table 2 and displayed in Fig. 1. The predicted population margin of MOUD receipt for non-Hispanic Black veterans with legal involvement was 32.9% (95% CI = 29.6–36.3%), for non-Hispanic American Indian Alaskan Native veterans without legal involvement was 38.2% (95% CI = 34.1–42.5%), for non-Hispanic White veterans with legal involvement was 43.7% (95% CI = 41.2–46.3%), and for non-Hispanic White patients without legal involvement was 42.1% (95% CI = 39.9–44.2%).

## Discussion

In a large national sample of veterans served at the VHA in Fiscal Years 2021–2022, non-Hispanic Black patients with legal involvement had lower odds of receiving MOUD compared to non-Hispanic White patients without legal involvement. These results suggest that for Black veterans, the interaction of racial/ethnic minority status and legal involvement can impact medication receipt. Results also indicated that non-Hispanic American Indian/Alaskan Native veterans without legal involvement had lower odds of receiving MOUD compared to non-Hispanic White patients without legal involvement. Interventions that target both racial and criminal legal barriers are needed.

Structural factors have been identified as barriers to MOUD for both legal involved and different racial/ethnically populations and may partially explain why Black veterans with legal involvement have the lowest receipt of MOUD. For example, Black people in the United States more frequently experience criminalization of opioid use compared to White people, and drug enforcement more commonly occurs in Black communities (James & Jordan, 2018), which may deter Black patients from seeking treatment and/or limit treatment access. Furthermore, Black people arrested for drug related charges have lower odds of being referred to, initiating, and graduating from a drug court than White people (Ho et al., 2018; Nicosia et al., 2013), suggesting Black people may have fewer opportunities to learn about or connect with treatment within legal contexts than White people and may be incarcerated more often limiting their ability to seek treatment at a VHA medical center. Although we do not have information on whether veterans in this study received medication during their incarceration, MOUD availability in prisons and jails is limited (Scott et al., 2021; Sufrin et al., 2023), which is a missed opportunity to educate about and initiate these medications for patients for whom it is indicated, particularly veterans from groups who are disproportionately incarcerated (Tsai et al., 2013). Even when MOUD is provided in carceral settings, White individuals are more likely to receive MOUD in jails than individuals from other racial and ethnic groups (The Commonwealth of Massachusetts, 2024).

The VHA may contribute to these structural factors through a lack of available services in geographic areas that serve more Black veterans or through policies, such as not honoring prison or jail prescriptions for MOUD for veterans who exit incarceration. Connecting patients to MOUD while in correctional settings increased treatment entry post-incarceration (Marsden et al., 2017), but care coordination between these settings and VHA is unknown. While there have been ongoing, successful efforts to improve prescribing of MOUD at the VHA (Hagedorn et al., 2022; Wyse et al., 2018), implementation efforts could inadvertently exacerbate or prolong differences in access if racial or criminal legal factors are not considered. Qualitive research, such as narrative stories (Mark et al., 2023), with Black veterans with legal involvement may shed light on why they have lower odds of receiving MOUD than their White veterans with legal involvement. Within the VHA, there is an effort to expand existing dashboards to monitor provision of MOUD by various patient factors may allow providers at each VHA to identify patients who would benefit from but are not utilizing MOUD. However, these dashboards would need to account for uncertainty due to small groups. A dashboard to successfully reduce racial differences in blood pressure control is one potential model that could also be used for patients who need MOUD (Burkitt et al., 2021).

Stigma and preferences for certain medications, both in the criminal legal system and in the healthcare system may limit available MOUD options (Andraka-Christou, 2021; Andraka-Christou et al., 2019; Hansen & Roberts, 2012; Lagisetty et al., 2019; Sufrin et al., 2023). Buprenorphine, which is more convenient than methadone, is less often prescribed to Black patients compared to White patients (Lagisetty et al., 2019). Federal regulatory differences between methadone and buprenorphine were driven by racial/ethnic associations (Hansen & Roberts, 2012). Practices that may reduce racial differences in MOUD include allowing office-based methadone without extensive regulatory barriers, increasing MOUD availability in primary care settings through a pharmacist care manager model, and improving permanent retention of remote buprenorphine induction via telehealth (Andraka-Christou, 2021; DeRonne et al., 2021). Criminal legal staff and healthcare provider training that addresses stigma associated with MOUD and race/ethnicity or legal involvement may also help improve gaps in medication receipt.

Interpersonal, systemic, and structural factors related to race may play a role in medication receipt (Braveman et al., 2022). Perceptions of an untrustworthy healthcare system, fewer race-concordant providers, and perceived discrimination from healthcare providers (Cuevas et al., 2016; King & Redwood, 2016; Saha et al., 2008) may result in Black patients seeking less care. Addressing differences in receipt of MOUD among non-Hispanic Black patients is especially critical because of the exponential growth in overdose rates among Black people (Jalal et al., 2018; Saloner et al., 2018). Non-Hispanic American Indian/Alaskan Native veterans without legal involvement also experienced a lower predicted population margin of MOUD receipt compared to non-Hispanic White veterans without legal involvement. Prior research with a general population sample indicates that patients who received methadone had higher odds of experiencing racial discrimination in a healthcare setting and that the odds of racial discrimination were especially pronounced among American Indian/Alaskan Native and Black patients (Pro & Zaller, 2020). Targeted and culturally appropriate outreach, particularly outreach conducted by peers (Giraldo et al., 2024), may address some of the perceptions Black veterans may have in seeking healthcare.

## Limitations

There were a few limitations to this study. First, MOUD that is accessed in non-VHA settings and paid for by the VHA are recorded in VHA data, but we have no ability to identify how many patients may seek care outside the VHA system that is not paid for by the VHA. Uncaptured treatment use may account for some of the differences we observed, such as use of non-VHA healthcare as a result of Medicaid expansion (Winkelman et al., 2016). Second, any patients with a record of being Hispanic were coded as Hispanic, regardless of race. Third, we were not able to determine if and where veterans were offered MOUD. Although prescription records indicate buprenorphine and naltrexone were offered at all facilities, we are unable to determine if all providers offer these medications or whether veterans received MOUD prior to their VHA treatment, such as during prison or jail incarceration. Finally, our legal involvement and race/ethnicity measures are limited. We cannot identify veterans who were reached out to by a Veterans Justice Programs staff member but did not respond or were unidentified as veterans in criminal legal settings; therefore, some veterans in the no legal involvement group may have had legal involvement. Furthermore, we do not have detailed information on veterans’ experiences with the legal system, such as the nature and severity of a veteran’s crime. Finally, we do not have other measures of race/ethnicity, such as perceived discrimination or implicit bias. Such measures could deepen our understanding of challenges patients experience in accessing MOUD (Mark et al., 2023).

## Conclusions

In this study, we provide evidence that differences in MOUD receipt are borne disproportionately by non-Hispanic Black veterans with legal involvement. VHA dashboards that stratify by various forms of marginalization may help to identify veterans who are not receiving MOUD. Interventions in specific criminal justice settings and efforts to reduce patient, clinical, and regulatory barriers to access may be needed to improve medication receipt among Black veterans with legal involvement.

## Data Availability

Data cannot be shared publicly because of VA policies regarding data privacy and security. Data contain potentially identifying and sensitive patient information.

## Abbreviations

AOR: Adjusted Odds Ratio
MOUD: Medications for Opioid Use Disorder
OR: Odds Ratio
US: United States
VA: Veterans Affairs
VHA: Veterans Health Administration
